# Driving forces of Antarctic krill abundance

**DOI:** 10.1126/sciadv.adh4584

**Published:** 2023-12-15

**Authors:** Alexey Ryabov, Uta Berger, Bernd Blasius, Bettina Meyer

**Affiliations:** ^1^ Alfred Wegener Institute Helmholtz Centre for Polar and Marine Research, Section Polar Biological Oceanography, Am Handelshafen 12, D-27570 Bremerhaven, Germany.; ^2^ Dresden University of Technology, Institute of Forest Growth and Computer Sciences, D-01062 Dresden, Germany.; ^3^ Institute for Chemistry and Biology of the Marine Environment, Carl Von Ossietzky University Oldenburg, Oldenburg, Germany.; ^4^ Helmholtz Institute for Functional Marine Biodiversity (HIFMB), Carl Von Ossietzky University Oldenburg, Oldenburg, Germany.

## Abstract

Antarctic krill, crucial to the Southern Ocean ecosystem and a vital fisheries resource, is endangered by climate change. Identifying drivers of krill biomass is therefore essential for determining catch limits and designating protection zones. We present a modeling approach to pinpointing effects of sea surface temperature, ice cover, chlorophyll levels, climate indices, and intraspecific competition. Our study reveals that larval recruitment is driven by both competition among age classes and chlorophyll levels. In addition, while milder ice and temperature in spring and summer favor reproduction and early larval survival, both larvae and juveniles strongly benefit from heavier ice and colder temperatures in winter. We conclude that omitting top-down control of resources by krill is only acceptable for retrospective or single-year prognostic models that use field chlorophyll data but that incorporating intraspecific competition is essential for longer-term forecasts. Our findings can guide future krill modeling strategies, reinforcing the sustainability of this keystone species.

## Teaser

Krill population model reveals how age-specific seasonal environmental factors and intercohort competition drive recruitment.

## INTRODUCTION

Climate change and ocean warming in the Southern Ocean are altering both the extent of seasonal sea ice and chlorophyll levels, leading to spatial shifts in marine ecosystems (*1*, *2*). Assessing climate-induced changes in the Antarctic ecosystem requires accurate modeling of Antarctic krill, the centerpiece of the food chain.

There is little consensus regarding the main factors determining krill abundance, which is perhaps expected given the extreme scales among relevant environmental and climate factors involved. Various studies indicate that krill survival can depend on ambient environmental factors such as chlorophyll concentrations (*3*, *4*), ice coverage (*5*, *6*), and water temperature (*7*) and on population factors such as reproductive density dependence (*8*) and competition with salps (*5*). However, since these local environmental factors fluctuate according to global climate (Box 1), krill recruitment has also been linked to climate indices such as the El Niño–Southern Oscillation (ENSO) and Southern Annular Mode (SAM) (*2*, *3*, *9*, *10*).

Box 1. **Planetary climate oscillations in the Southern Hemisphere.**

**El Niño Southern Oscillation**

ENSO is an irregular fluctuation in temperature and pressure in the tropical Pacific region. These fluctuations occur every 2 to 5 years and can have strong impacts on global weather patterns. ENSO has two main phases: El Niño, which is characterized by warm temperatures in the eastern Pacific, and La Niña characterized by cool temperatures in this region. ENSO events are characterized by the Southern Oscillation Index (SOI), which measures air pressure variations in the surface layer of the atmosphere between eastern and western Pacific waters, and by the Niño3.4 index (see Materials and Methods), showing sea surface temperature anomalies in the equatorial Pacific Ocean in the so-called Niño 3.4 region.

The mechanisms driving ENSO are not fully understood, but the transition is often linked to the strength of the trade winds (tropical winds blowing steadily from east to west toward the equator). Trade winds drive the North and South Equatorial Currents, which transport both cold upwelling waters and the cold-water Humboldt Current (which originates in the Southern Ocean) from east to west. As these surface waters move westward, they warm, causing the western Pacific to become warmer than the eastern Pacific. During La Niña years, the trade winds are strong, resulting in temperatures in the western Pacific that are around 8° to 10°C warmer than in the eastern Pacific. On the other hand, during El Niño years, the trade winds and ocean currents weaken and sometimes reverse, leading to a decrease in upwelling and a substantial warming of the tropical eastern Pacific (*60*).

El Niño causes an anomalous anticyclone (counterclockwise outspiralling wind) in the southeast Pacific with warm north winds that heat the ocean and reduce ice levels in the Amundsen, Bellingshausen and Ross seas and south winds that cool the ocean and increase ice levels in both the South Atlantic (Weddell Sea) and Antarctic Peninsula (*61*). In contrast, a cold La Niña period causes a cyclone (clockwise wind spiral) that leads to a cooling of the South Pacific and warming of the Antarctic Peninsula and the South Atlantic. This phenomenon is called the Antarctic Dipole (*62*).

**Southern Annular Mode**

SAM, also known as the Antarctic Oscillation (AAO), describes the latitudinal shift of the belt of westerly winds circulating around Antarctica and governs the distribution of precipitation and temperature from the subtropics to Antarctica (*63*). The positive phase of SAM corresponds to stronger westerly winds over middle and high latitudes (50° to 70°S) and weaker westerly winds in mid-latitudes (30° to 50°S). Temperatures and chlorophyll concentrations around the Antarctic Peninsula are positively correlated with the current SAM index (*45*). However, the concentration of large diatoms, the preferred prey of adult krill, may decrease during positive SAM phases (*3*). This mode is characterized by Marshall’s SAM index, which is based on the zonal pressure difference between 40° and 65°S or, correlated with it, the AAO index calculated by the National Oceanic and Atmospheric Administration based on isobar height anomalies poleward of 20°S, which is considered to be the best indicator of SAM behavior (*64*).

An additional source of uncertainty for krill modeling stems from the fact that field results are often presented as correlations between, for instance, driving factors and recruitment, which do not capture the dynamics of underlying functional relationships nor the relative strength and timing of the different drivers. Therefore, mechanistic models of krill often rely on krill traits, such as consumption or growth rate, which have been measured under steady-state laboratory conditions. Moreover, traits such as egg production, hatching, and starvation mortality are not fully studied and are typically estimated using allometric scaling models (*8*, *11*, *12*). As a consequence, the mechanisms of density regulation of reproduction are also poorly understood.

The dependence of recruitment on the number of producers can be described by the phenomenological Beverton-Holt (BH) model (*13*), which suggests that the number of larvae increases monotonically with the number of adults to a level determined by the maximum carrying capacity of larval habitats, e.g., under winter sea ice (*14*, *15*), where they are not affected by competition with adults. Ricker (RK)’s alternative model suggests that reproduction begins to decline because of intra- and intercohort competition as the number of adults exceeds a critical level (*16*, *17*). The RK model has been successfully applied to describe fluctuation in abundance of insects (*18*) and fish (*19*) and analyze intraspecific competition (*20*, *21*). Considering intercohort competition for a shared resource may explain krill oscillations (*8*), but there is currently no firm indication for or against this theory (*22*). A systematic test of this theory by a modeling study could support or refuse its plausibility.

We aim to identify the environmental factors that control krill recruitment and survival, and to analyze the dependence of recruitment on adult density by comparing krill dynamics that are simulated by the BH and RK models. We first fit models with time-constant parameters and then find minimal annual deviations (anomalies) of these parameters at which the model best reproduces fluctuations in the abundance of juveniles and adult krill. Investigating links between the anomalies and environmental factors using machine learning techniques allows us to determine the underlying drivers of krill dynamics and to develop an environmental-driven model that closely approximates observed krill dynamics.

## RESULTS

### Data

**Fig. 1.**
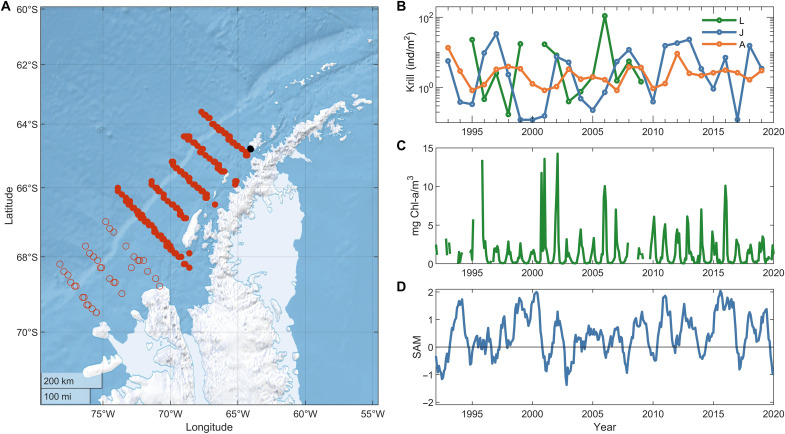
Dynamics of krill abundance and driving factors. (**A**) PAL-LTER grid station locations for summer krill sampling (red circles). Only filled circles (center and north lines) were used to parameterize the model. The black circle shows the location of regular conductivity, temperature, and depth data sampling stations: B (coastal; depth ≈ 75 m) and E (offshore; depth ≈ 200 m). (**B**) Average abundance of larval (green), juvenile (blue), and adult (orange) krill. Note that larval abundance was measured in the AMLR grid north of the Palmer grid. (**C** and **D**) Dynamics of chlorophyll concentrations (Chl-a; green) and SAM index (blue).

Summer postlarval krill abundance data at the Western Antarctic Peninsula (WAP) were collected over 27 years as part of the Palmer Long-Term Ecological Research (PAL-LTER) program *(**4*, *9*). The Palmer program’s sampling grid (Fig. 1A), approximately 600 km by 200 km in size, was selected because of the effect of its local gyres, which tend to retain the krill population and minimize advective emigration and immigration (*23*). We used data from the northern and central parts of the grid (filled circles) because the krill population in these areas was monitored throughout the entire period and had coherent spatial population dynamics (*9*). Unfortunately, the PAL-LTER data do not include larval abundances. To adjust the reproductive functions in our models, we supplemented our dataset with larval abundance in January to February (*10*) based on data from Antarctic Marine Living Resources program (AMLR) (*24*). Although AMLR stations are located around 500 km north of the Palmer stations at the North Atlantic Peninsula, the dynamics of both populations are highly correlated (*25*). We used only a portion of the AMLR data because the AMLR grid area is not a relatively closed system like the Palmer grid region.

Over the 27-year monitoring period, the total abundance of postlarval krill fluctuated by more than two orders of magnitude, which is reflected in the fluctuations in larvae, juvenile (≤35 mm), and adult (body size >35 mm) abundances (Fig. 1B). Four large 4 to 5 year cycles were followed by a period when the abundance of adults was almost stable from 2013 to 2019. Similar cycles are observed in krill size distribution dynamics, where 2 years of successful reproduction led repeatedly to cohorts that dominated the population for 4 to 5 years (*3*, *8*). These cycles also correspond to larval reproduction peaks in the AMLR data (green line, Fig. 1B) and are likely related to variability in environmental factors (e.g., chlorophyll a; Fig. 1C), which, in turn, are driven by global climate anomalies (e.g., SAM; Fig. 1D).

### Life history labyrinth model

#### Time-constant model

**Fig. 2.**
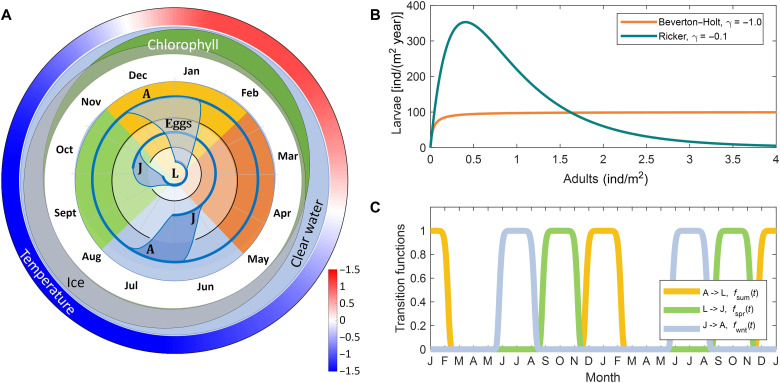
The krill reproduction cycle. (**A**) Schematic representation of the krill life history labyrinth model. The inner circles show the different functional stages: larvae (L), juveniles (J), and adults (A). The life cycle (blue line) starts from the center of the labyrinth, rotates clockwise with the season, and spirals outward when the transition is open (black lines represent the walls of the labyrinth); different seasons are labeled with different colors (for example, summer is yellow). The outer circles show the mean values of chlorophyll concentrations (green), temperature (red-blue color map), and ice coverage (gray) in Palmer grid. (**B**) Generalized Deriso-Schnute stock-recruitment function (see Materials and Methods). In this function the control parameter γ defines a smooth transition from the BH model (orange, γ = −1) to the RK model (green, γ → 0). (**C**) Functions controlling the activation of the transition windows between krill stages, plotted over the course of 24 months.

Krill population dynamics can be schematically represented as the movement of cohorts along a spiral labyrinth of life history (Fig. 2A), where transitions from an inner ring to a functionally different stage on the next ring occur only during certain time periods. We distinguish the following three krill cohorts (*26*). Larvae appear in summer and are not only the most abundant but also the most vulnerable group to starvation. Juveniles include individuals older than 9 months that externally look like adults but are not yet ready for spawning. The last group consists of adult, sexually mature krill at least 2 years old. The seasonality of the krill life cycle is driven by annual variations in sea surface temperature, chlorophyll concentrations, and ice coverage, whose relative values (averaged over the observation period) are presented in the outer ring. Cohort abundances increase via summer reproduction (L) and recruitment from the previous developmental stage (J and A). A decrease in cohort abundance may be caused by mortality or transitions to the next stages.

The population dynamics comprises reproduction with rate *R*(*A*) (larvae), mortality at rates *m*_L_, *m*_J_, and *m*_A_, and time-dependent transitions between stages at maximal rate *r* that occur when the activating transition functions (Fig. 2C) approach 1. The dynamics of cohort abundance can be described by the following set of differential equations

$$\frac{d}{dt}L=R(A)f_{\mathrm{sum}}(t)-{rf}_{\mathrm{spr}}(t)L-m_{L}\;L$$

$$\frac{d}{dt}J={rf}_{\mathrm{spr}}(t)L-{rf}_{\mathrm{wnt}}(t)J-m_{J}\;J$$

$$\frac{d}{dt}A={rf}_{\mathrm{wnt}}(t)J-m_{A}A$$

We assume that larval reproduction, *R*(*A*), follows the Deriso-Schnute function (Fig. 2B), in which the parameter γ specifies a smooth transition from a BH model (γ = −1, BH model), where the maximum number of larvae increases monotonically with adult abundance, to a RK model (γ → −0, RK model), where an increase in adult population above a threshold value reduces the number of survived larvae, implicitly modeling competition for a shared resource (see Materials and Methods for the detailed description). We refer below to this model with time-independent parameters as a “time-constant” model.

#### Fitting procedure, time-constant model

To understand the mechanisms driving krill population dynamics, we propose to use a three-step modeling approach. In the first step, we describe krill dynamics using models with time-constant parameters, where krill recruitment can only vary with the abundance of adult krill. In the second step, we account for environmental interannual variability by determining annual anomalies (deviations) in the mortality of larval and juvenile krill. Last, by analyzing the relationship between these anomalies and environmental factors, we can identify and compare the key drivers of krill population dynamics for both models.

**Fig. 3.**
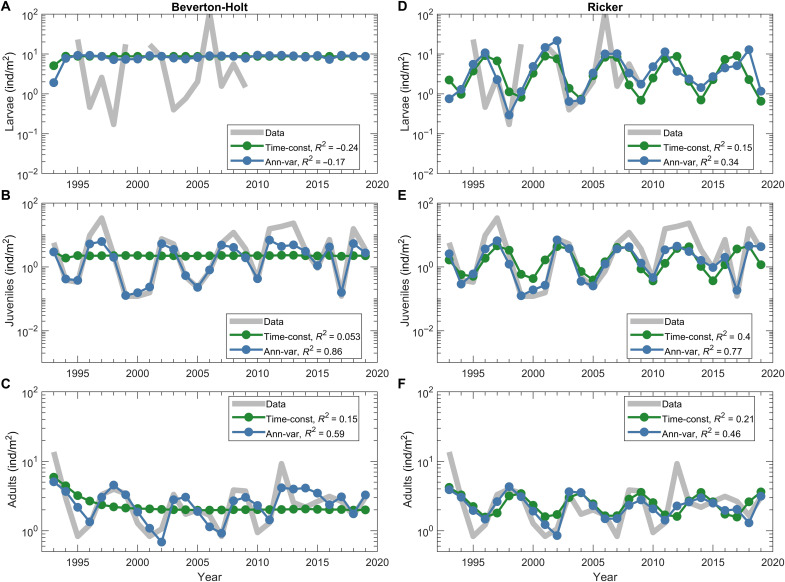
Fitting models to data. Larvae (**A** and **D**), juvenile (**B** and **E**), and adult (**C** and **F**) abundances obtained from krill sampling and modeled with the BH model [(A) to (C)] and RK model [(D) to (F)]. Shown are mean krill abundance (gray), abundance from time-constant models (green), and abundance from models with annual variability of larvae and juvenile mortalities (blue). To match the data here and below, we show model outputs on 1 January. Note that larval abundance was measured in the AMLR grid north of the Palmer grid, while juvenile and adult abundance from PAL-LTER data.

The best fits of the time-constant models are shown in Fig. 3. The BH model cannot reproduce population oscillations and predicts population stabilization at an average level, regardless of initial conditions (Fig. 3, A to C, green line). Note also that the best fit resulted in a relatively steep initial slope of the reproduction function (Fig. 2B, orange line). In contrast, the RK model gives a good approximation of larvae, juvenile, and adult abundance oscillations over about 20 years but loses synchronization with the data in the last third of the observation period (Fig. 3, D to F, green line). The discrepancy between both models and data occurs because the models do not account for interannual climate variability, so these results can only be interpreted as an expected abundance of krill under unchanging year-to-year climate conditions.

#### Model with annually varying mortality

To account for climate variability, we introduce annual anomalies of losses. We define an anomaly of loss in cohort *k* and year *Y* as a coefficient δ_*k*,*Y*_ such that the mortality in year *Y* equals $m_{k,Y}^{\prime }=m_{k}$ exp δ_*k*,*Y*_, where *m_k_* is the standard cohort mortality (Table 1). Thus, the loss rate *m_k_* characterizes the background level of losses from factors such as aging, fishing, and predation that are either constant over time or that we do not have sufficient data to analyze (predation). By contrast, anomalies characterize deviations from the background level in year *Y* favorable (δ_*k*,*Y*_ < 0) or unfavorable (δ_*k*,*Y*_ > 0) for that cohort. Thus, we include interannual variations only in the mortality, while the other parameters, e.g., the reproduction function, remain the same as in the time-constant model. Therefore, the larval mortality anomalies should be viewed as effective parameters reflecting variations in both mortality and larval reproductions.

**Table 1.** Parameters of the time-constant RK and BH models. Parameters were obtained by minimizing the weighted SD between the logarithms of modeled and field-estimated adult, juvenile, and larval abundances (Fig. 3, gray and green lines). Weights (*w*_L_ = 2/3, *w*_J_ = 1, and *w*_A_ = 2) were chosen such that each cohort contributed equally to the overall SD.

	BH	RK	Units	Meaning
**Preset model parameters**				
γ	−1	−0.09		Controlling transition from BH to RK model
*r*	30	30	Year^−1^	Transition rate between stages
τ	0.2	0.2	Year	Duration of the window (Fig. 2C) when the transition between stages is allowed
**Fitted standard parameters of the time-constant model**				
*L*_max_	3368	2481	Eggs/year per ind	Maximal number of larvae produced by one adult per time unit
*G*_max_	101	353	Eggs/year per square meter	Maximal number of larvae per square meter per time unit
*m*_L_	2.6	1.76	Year^−1^	Larval mortality
*m*_J_	1.31	1.13	Year^−1^	Juvenile mortality
*m*_A_	0.6	0.51	Year^−1^	Adult mortality
**Initial conditions on 1 January 1993**				
*L*_0_	1.9	0.75	Ind/m^2^	Larvae
*J*_0_	2.94	2.6	Ind/m^2^	Juveniles
*A*_0_	5.07	3.9	Ind/m^2^	Adults

**Fig. 4.**
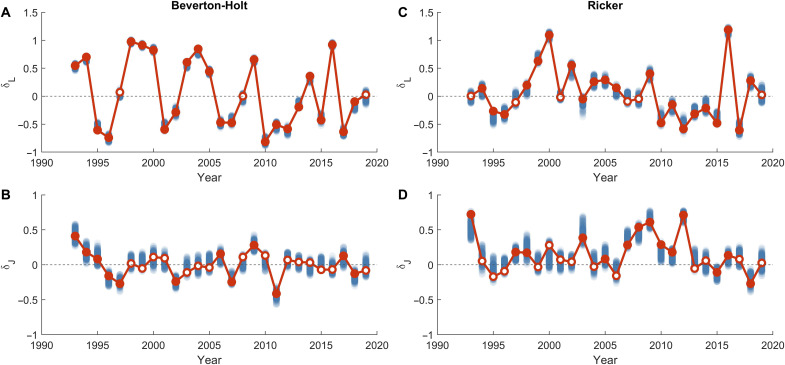
Anomalies of larvae and juvenile losses. Minimal annual anomalies of larval (**A** and **C**) and juvenile (**B** and **D**) losses in the BH (A and B) and RK (C and D) models that provide best fits of the annually varying model to data. Blue dots show anomalies resulting from 300 reruns of the parameter optimization cycle. The red dots indicate the anomalies yielding the best fit across these cycles (solid circle for significant and open circle nonsignificant anomalies); these anomalies were used for the annually varying models in Fig. 3.

Determining the loss anomalies is a complex task of searching for a global minimum in a high-dimensional space. To avoid model overfitting and to reduce outliers among loss anomalies that could arise, for example, from possible krill abundance measurement errors, we searched for loss anomalies that provided the best fit between the model and the data but had the minimum sum of their squares (see Materials and Methods). To test the robustness of our results, we ran 300 minimization cycles. Anomalies from the minimization cycle that provided the closest fit of the model to the data are shown by red dots in Fig. 4, anomalies from other cycles are shown by blue dots. Across all cycles, the model fit remained virtually unchanged (less than 2% variation in the model to data deviation), which allows us to interpret the spread of the blue dots in Fig. 4 as a confidence interval. We consider an anomaly significant (filled circle) if at least 95% of the blue dots are on the same side of zero; otherwise, it is considered insignificant (open circle). On the basis of this analysis, we excluded adult loss anomalies because most of them were insignificant.

The introduction of annual loss anomalies substantially improves the fit of both models to the data (Fig. 3, blue lines). Note that in the BH model, larval abundance remains steady (Fig. 3A) because its 1 January level is primarily influenced by the reproduction function and because the adult abundance largely varies within the saturation region of the stock-recruitment function (orange line in Fig. 2B). However, the annual deviations in larvae losses translate the nearly constant larval abundances into oscillations in juvenile and adult populations, aligning the model output with the data. In contrast, the reproductive function in the RK model directly translates oscillations in adult abundance into oscillations in larval abundance (Fig. 3D). Therefore, it is expected that the larval loss anomalies in the RK model are different from those in the BH model.

The anomalies of losses obtained in the RK model and the BH model do not correlate with each other, suggesting that they are likely related to different environmental drivers (Fig. 4). In both models, larval loss anomalies are larger and more precisely defined than juvenile anomalies. For larvae, there are groups of consecutive years where loss anomalies were significantly positive (high losses) and negative (low losses). In contrast, about half of the juvenile loss anomalies differ only insignificantly from zero (compare Fig. 4, A and C and B and D). Thus, we can expect that environmental variability influences the larval population stronger than the juvenile population.

### Statistical analysis

#### Correlations between annual loss anomalies and environment

**Fig. 5.**
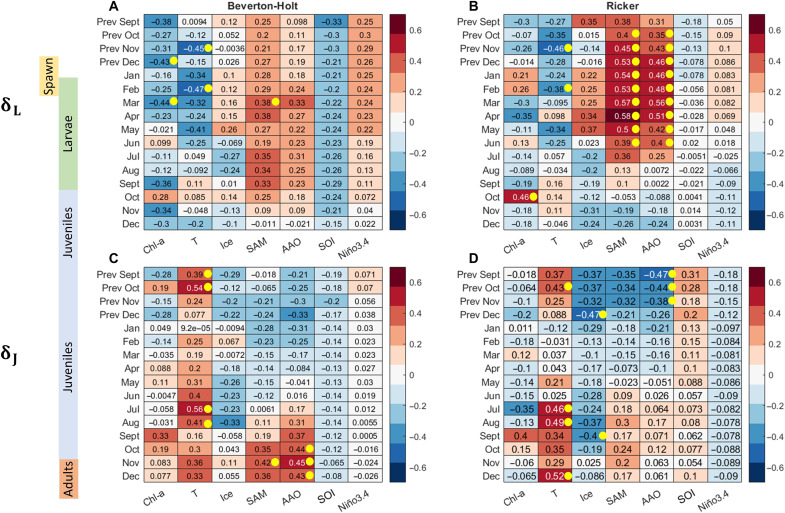
Effects of the environment on krill losses. Pearson correlations between average values of environmental factors and annual anomalies of larval (**A** and **B**) and juvenile (**C** and **D**) losses were calculated for the BH [(A) and (C)] and RK [(B) and (D)] models. Rows in each plot correspond to the month in which a factor was measured, and columns present the factor itself. Monthly averages were used for chlorophyll concentrations, temperature, ice coverage, and a 9-month moving average for the climate indices. The bar on the left side roughly shows the stages of krill development in each month. Yellow dots indicate significant correlations (*P* < 0.05).

To link the obtained anomalies with environment, we considered their correlations with monthly average values of environmental factors and climate indices (Fig. 5). We use monthly averaged chlorophyll concentrations (Chl-a) and sea surface temperature (*T*), regularly measured at stations B and E (Fig. 1A), and satellite estimates of ice coverage in the Palmer grid region (Ice). As global climate characteristics, we rely on four indices: two related to the ENSO, namely, the Southern Oscillation Index (SOI) and the Niño 3.4; in addition, the Antarctic Oscillation (AAO) and the closely related SAM index. See Box 1 and Materials and Methods for more details. As these indices change rapidly and determine water temperature and ice conditions in WAP with a lag, they are usually replaced by a moving average with sampling window of 6 to 12 months (*7*, *10*). We used 9-month moving averages to match the analysis by Atkinson *et al.* (*7*), which demonstrated that average SAM anomalies from January to September predict recruitment in the following summer. To evaluate the influence of anthropogenic factors, we also examined the correlations between loss anomalies and fishery data in this area (*27*). However, this analysis did not reveal any statistically significant correlations. Note that the absence of these correlations does not exclude the presence of an effect because fishery efforts in this region display a relatively small year-to-year variability, changing rather on decadal scales, and therefore their effect is difficult to capture with correlation analysis.

The larval loss anomalies obtained for the BH model show a negative correlation with chlorophyll concentrations and temperature at the beginning and end of the summer period and a negative correlation with SOI during the spring maturation to spawning period and positive correlations with the SAM index (Fig. 5A). In contrast, larval loss anomalies in the RK model are not correlated with chlorophyll, but we observe a block of significant positive correlations both with SAM and with AAO from October to June, as well as negative correlations with temperature in November and February (Fig. 5B). In both models, a decrease in larval losses aligns with an increase in ice cover during late winter (negative correlations in July and August).

Both models indicate that environmental factors that have a positive impact on one developmental stage may have no effect or a negative effect on the other stage, suggesting a niche partitioning between these two krill stages. For example, expanding ice cover and lower temperatures in spring, fall, and winter can reduce juvenile losses (Fig. 5, C and D), but lower temperatures in spring and summer can increase larval losses (Fig. 5, A and B).

Climate indices also exhibit opposing correlations with larval and juvenile survival. These correlations are stronger in the RK model, but the BH model reveals similar trends. Specifically, both AAO and SAM display a significant positive correlation with larval losses in RK model, while these indices during the period from September to November demonstrate negative correlations with juvenile losses. Thus, a negative SAM and AAO phase correspond to the success of larvae reproduction and survival, while the success of juvenile stage can be associated with a positive phase of these indexes. Correlations with SOI and Nino 3.4 demonstrate similar patterns, but these correlations are much weaker. Nevertheless, they align with observations made by Loeb and Santora (*10*), who associated negative Nino 3.4 with larval survival and positive Nino 3.4 with the successful transition to the juvenile stage.

#### Disentangling environment effects on krill survival during their first 2 years

To reconstruct a continuous time pattern of specific environmental effects on krill loss anomalies during the first 2 years of their life cycle, we applied a machine learning technique called fused least absolute shrinkage and selection operator (LASSO) regression (*28*). On the basis of correlation analysis, we identified chlorophyll, *C*, temperature, *T*, ice coverage, *I*, and SAM, *S*, as potential drivers of anomalies. All variables were standardized to zero mean and unit variance, and we represented the loss anomalies for cohort *k* and year *Y* as the sum of factors over consecutive months

$$\delta_{k,Y}=a_{0}^{k}+\sum_{i}a_{i}^{k,C}C_{Y}^{i}+\sum_{i}a_{i}^{k,T}T_{Y}^{i}+\sum_{i}a_{i}^{k,I}I_{Y}^{i}+\sum_{i}a_{i}^{k,S}S_{Y}^{i}$$ (1)

where the index *i* spans 16 months (from September to December of the next calendar year, as shown in Fig. 5), and the coefficient $a_{i}^{k,F}$ characterizes the impact of factor *F* on losses in cohort *k* during month *i*.

**Fig. 6.**
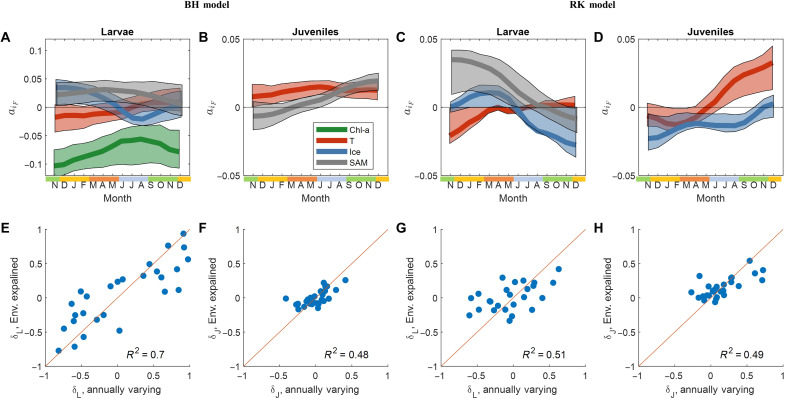
Forecasting of loss anomalies based on environmental factors for the BH and RK models. (**A** to **D**) Solid lines show coefficients *a_i_^F ^*in Eq. 1 selected by fused LASSO regression for the effects of chlorophyll (green), temperature (red), ice (blue), and SAM (gray) on losses of larvae and juvenile krill. Shading shows 90% confidence intervals calculated by fused LASSO regression for bootstrapped data sequences. (**E** to **H**) Comparison of larval/juvenile loss anomalies fitted from the annually varying model (red circles in Fig. 4A) and those predicted by Eq. 1 with the coefficients shown in (A) to (D).

This linear model encompasses four factors measured over the 16-month span, totaling 54 variables. In these cases, traditional linear regression often tends to overfit the data, leading to suboptimal performance on unseen datasets. To address this, we need to reduce the model’s degrees of freedom. Fused LASSO offers an effective approach for extracting maximum information from small datasets with a large number of variables (*29*), when we can assume that closely related variables, like consecutive monthly chlorophyll levels (e.g., in December and January), have similar effects on the target variable. Thus, the difference between adjacent coefficients, $\left(a_{i}^{k,F}-a_{i+1}^{k,F}\right)$, should be minimal. Unlike traditional feature selection methods, fused LASSO retains correlated variables, such as consecutive monthly chlorophyll values, while aiming for a sparse linear model with minimal differences between adjacent coefficients. In Materials and Methods, we provide a detailed description of this algorithm, describe the hyperparameter selection, and evaluate the relative importance of different factors (fig. S1). The coefficients $a_{i}^{k,F}$ of the selected models are shown in Fig. 6 A to D.

First, we consider the dependencies of anomalies on environmental factors common to both models. Both the BH and RK models indicate that from late spring to autumn (spawning and larval development), increasing temperature and decreasing ice coverage reduce larval losses (Fig. 6, A and C). However, in winter, the pattern reverses, and larval losses are reduced with increasing ice, emphasizing ice’s protective role for overwintering. Simultaneously, the variations in water temperature seem to have little to no effect on larval losses in winter.

The reduction of larval losses with increasing temperature has several mechanistic explanations. First, during spring and summer, warmer temperatures boost phytoplankton productivity (*30*). Second, an increase in temperature from −2° to 2°C might accelerate embryo and early-stage development (*31*, *32*). However, this is valid for Palmer-grid region only, where summer temperatures stayed below 2°C, while higher temperatures have a strong negative impact on hatching success and embryo development (*33*).

The impact of strong winter with long and intense ice coverage until late spring/early summer on krill spawning success and larvae survival is debated. Our findings suggest that late ice retreat might reduce recruitment success. This challenges older views that a late ice retreat promotes early krill spawning and favors recruitment (*3*, *34*). Instead, our results align with studies indicating that krill reproduction success is favored by moderate winter before spawning and moderate spring ice conditions (*6*, *23*, *35*).

In contrast to in summer, both models predict that the effects of temperature and ice on krill larval survival are reversed in winter. Larval krill losses in winter decrease with increasing ice coverage (both models) and increase with increasing temperature (BH model). This aligns with the hypothesis that strong winter with large sea ice extent promotes higher krill larvae survival (*23*). Pack ice serves as a vital nursery habitat for krill larvae (*36*), offering complex under ice structures that afford protection from predators (*15*). Sea ice biota and the associated zooplankton community are essential food sources (*37*), which, in some cases, fully cover the winter energy budget (*38*).

The main difference between the BH and RK models lies in the most critical factors linked with larval losses. In the BH model, chlorophyll concentration plays a central role, consistently reducing larval losses. Its impact is most pronounced in late spring and summer during female maturation, spawning and larval weight gain in summer and early autumn. The impact of chlorophyll reduces in winter and then increases again in the next spring during the transition from the larval to the juvenile stage (Fig. 6A). In contrast, the RK model reveals that larval losses increase with a rising SAM climatic index (Fig. 6C). This relationship is the strongest in spring and summer and gradually decreases as larvae approach the juvenile stage the following spring.

The mechanistic effects of chlorophyll on recruitment (Fig. 6A) is multifaceted, as phytoplankton is an important component of the krill diet (*39*, *40*), influencing reproduction, growth, and development (*14*, *41*, *42*) as well as krill lipid levels (*43*). Although krill is mainly considered omnivorous, this mainly comes into play in seasons when phytoplankton biomass is low, such as in autumn and winter (*44*). Therefore, warmer summer temperatures, with increased chlorophyll levels and associated zooplankton biomass, can cause favorable feeding conditions for krill larvae and facilitate larval development to advanced stages with sufficient lipid reserves for overwintering (*37*). Furthermore, high food availability in autumn may reduce competition between cohorts of different ages (*8*). In late winter and early spring, late larvae stages require abundant phytoplankton to meet their energy demands for rapid growth and transitioning to the juvenile stage (*15*). These findings also align with the observation that recruitment peaks in WAP region are linked to chlorophyll anomalies in the preceding year (*3*).

The positive correlation between SAM and larval losses in the RK model (Fig. 6C) can also be explained mechanistically. The largest regression coefficients (Fig. 6C) and highest correlations (Fig. 5B) are found between larval losses and 9-month averages of SAM values for November to March of the following year. All of these 9-month moving average values encompass the prespawning overwintering period. Thus, given that positive SAM values are typically associated with warmer temperatures (*45*), an increase in larval loss corresponding to increases in these averages suggests that increased temperature during the prespawning overwintering period may negatively affect the abundance of adult krill matured for spawning in the spring. Note that the SAM effect we identified occurs at an earlier time point compared to the results of Atkinson *et al.* (*7*). The average January to September SAM index they use coincides with our moving average in September. As shown in the regression (Fig. 6C) and correlation (Fig. 5B) analyses, this value does not show a significant link with krill losses. This discrepancy can be explained by the fact that the Palmer grid is located south of the 60° to 62.5°S belt included in the study of Atkinson *et al.* (*7*).

**Fig. 7.**
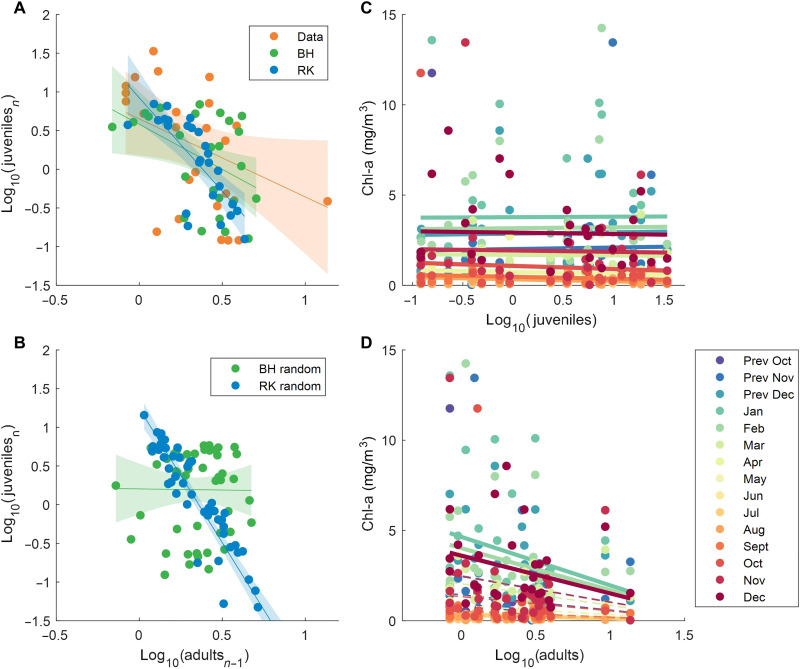
Negative effect of adult population on chlorophyll and recruitment. (**A**) Relationship between the adult abundance and juvenile abundance obtained in the following year for the data and annually varying BH and RK models (see Fig. 3). (**B**) Comparison of BH and RK models results with randomly permuted loss anomalies. (**C** and **D**) Effect of juveniles and adults on mean chlorophyll concentrations in different months before and after abundance sampling. Thick lines show significant relationships (*P* < 0.01) as identified by the linear mixed-effects model, where months of chlorophyll observations were considered as random effect terms.

The dependence of juvenile losses on environmental factors exhibits similar trends in both models (Fig. 7, B and D). Losses rise with increasing temperature (in the BH model throughout the year and in the RK model during winter and spring). In addition, losses increase with decreasing ice coverage in the RK model. Notably, temperature and ice display a negative correlation, indicating a close inverse relationship between these factors. These findings support hypotheses suggesting that elevated temperatures can have adverse effects on subadult and adult krill (*2*, *7*) and that during winter, high ice coverage, offering shelter and food, benefits both juvenile and larval survival. Specifically, in autumn, late juvenile krill can accumulate essential omega-3 fatty acids by feeding on sea ice biota, facilitating their maturation into the adult stage (*46*).

Overall, environmental factors explain approximately 70% of the larval loss anomalies in the BH model and about 50% of the loss anomalies in other cases (Fig. 6, E to H). Using these relationships, we parametrized environmentally driven models for krill population dynamics, wherein annual loss anomalies were determined by the corresponding linear dependences on environmental factors (fig. S2). With this parameterization, both the RK and BH models could account for approximately 60% (the RK model) to 70% (BH model) of the variation in juvenile abundance and yielded comparable predictions for adult abundance (*R*^2^ = 0.45 for BH model and *R*^2^ = 0.42 for the RK model).

#### Chlorophyll, SAM, or both?

Therefore, both models, when driven by environmental factors, can provide comparable predictions of krill dynamics. However, the BH model suggests that larval survival is primarily linked to chlorophyll, while the RK model shows that larval survival depends on the SAM index. Although both chlorophyll and SAM can be connected to recruitment, the question remains as follows: Why does dependence on chlorophyll appear in the BH model, which does not explicitly account for between-cohort competition, while dependence on SAM appears in the RK model, which does consider such competition?

We believe that both results can be explained within a single framework, assuming that chlorophyll is a shared resource and its concentration depends not only on climatic factors but also on krill biomass. Since the BH model does not account for the feedback between resource (chlorophyll concentrations) and consumers (krill), chlorophyll concentrations appear to be the main driving factor in it. However, this prediction only works as long as we know the in situ chlorophyll concentrations in the presence of krill. In contrast, the RK model already accounts for competition between cohorts for a shared resource and shows that larval loss anomalies are linked to a climatic index, SAM, independent of krill biomass. AAOs affect temperature, sea ice extent, and primary production, so they can determine variations in the carrying capacity of krill habitat and adult productivity, which manifest in the RK model as annual deviations of larval losses from the time-constant model parameters.

This hypothesis is supported by the fact that in PAL-LTER region chlorophyll concentrations in spring and summer decrease with increasing adult abundance, which is characteristic of top-down control systems (Fig. 7D). Furthermore, in both the data and models, the number of recruits is negatively correlated with the number of adult krill (Fig. 7A). This relationship naturally occurs only in the RK model and requires a specially tailored sequence of larval losses in the BH model. To test this hypothesis, we conducted model runs in which the sequence of loss anomalies, shown in Fig. 4, was randomized to mimic an arbitrary sequence of favorable and unfavorable years. In this case, for the BH model, we obtained a neutral dependence of recruits on the number of adults, while the RK model still resulted in a negative correlation between adults and recruits (Fig. 7B).

## DISCUSSION

To examine the driving forces of krill abundance, we introduce a modeling approach consisting of three stages. First, we fit a system of differential equations with time-constant parameters to species abundance data, creating a null model representing the system dynamics’ baseline. Second, we identify the minimum annual deviations (anomalies) in model parameters that optimize the model fit to the data. Third, we analyze how these anomalies depend on environmental factors. This approach is parameter-free, as it avoids defining specific functional relationships between model parameters and environmental factors, particularly given the often complex and poorly understood nature of these dependencies, typically defined only under controlled laboratory conditions. Instead, in the final step, we derive “field” functional dependencies of anomalies on environmental factors.

We determine loss anomalies by minimizing both the deviation of the model from the data and the variance of anomalies across the entire dataset. Compared to traditional approaches, where fluctuations in recruitment are estimated from adult and juvenile abundances in two consecutive years (*3*, *10*), our method offers more robust estimations, effectively suppressing potential outliers. Traditional methods can be likened to finding the slope of a line passing through only two experimental points, while our approach is closer to a least-squares fit of a function generated by the dynamic model to all data points. In the traditional approach, errors in each calculation are primarily influenced by the uncertainty in krill abundance in the two selected years, neglecting the impact of prior and subsequent population abundances. In contrast, fitting annual anomalies in our approach relies on the entire dataset, thus accommodating possible model feedback and reducing uncertainties. Our approach is close to the one used by Kinzey *et al.* (*22*) for determining krill recruitment. However, we take an additional step by linking anomalies to environmental conditions, enabling us to quantify the effects of environmental factors on krill survival and parameterize predictive population models.

Our life history labyrinth model blends iterative models with time-continuous models, yielding two key advantages. First, being based on differential equations, our modeling approach allows us to predict the krill population continuously throughout the year. This flexibility simplifies integration of factors influencing krill populations during specific seasons and estimation of krill abundance on any given day. This is an advantage over iterative models, which typically provide krill biomass estimates for isolated time points, e.g., in mid-summer (*6*, *22*), making it challenging to incorporate processes that vary continuously throughout the year, such as fisheries impact, predation, ocean currents, etc. Second, the use of predetermined time windows for transition between krill developmental stages makes our model relatively insensitive to the rate of this transition (see Materials and Methods). As transition windows between stages do not overlap, our model is free from artifacts such as the instantaneous transition of a fraction of larvae to the adult stage immediately after spawning, which potentially arise in typical differential equation-based models.

Our study synthesizes many previous findings, indicating that krill recruitment is a complex process jointly influenced by chlorophyll concentrations, temperature, ice, and SAM index, and their effects change with season and krill stage. In addition, we conjecture that fluctuations in krill biomass are likely the result of competition between adults and larvae for shared resources. This is demonstrated by the fact that the anomalies observed in the RK model, which accounts for intercohort competition, do not depend on chlorophyll concentrations. In contrast, the best fit of the BH model, which does not account for this competition, requires the modulation of the model by local chlorophyll values, which, in turn, are not independent variables, but can be negatively affected by krill populations. Therefore, the interspecific competition included in the RK model is manifested through the dependence of resources on adult abundance in the BH model. This result supports our earlier finding that cycles of krill biomass are driven by intercohort competition (*8*).

Substantial top-down control of resources by krill is supported by earlier studies. First, Loeb *et al.* (*5*) note significant negative local correlations between phytoplankton and abundance of krill and salps. Second, the peak of the dependence of larvae losses on chlorophyll fluctuations occurs in December (Fig. 6A), coinciding with the maximum grazing impact of krill (*47*). Third, although larvae and adults usually occupy distinct habitats (*48*), they can still compete for the same resources. This can be seen in populations of salps and krill, which also lack spatial correlation (*49*), but their abundances show that an inverse relationship and competition for resources are two of the potential hypotheses (*4*, *5*). Last, studying krill top-down control on phytoplankton through simple correlation analysis poses challenges due to krill actively seeking feeding grounds with higher chlorophyll concentrations, resulting often in a positive correlation between krill abundance and local chlorophyll concentration (*50*). A comprehensive investigation would require an experiment comparing the balance of particulate organic carbon, primary productivity, krill grazing rate, and carbon bound in krill fecal pellets and microbial recycling loop. In addition, one should take into account sinking and diffusion rates of fecal pellets and phytoplankton.

Consequently, we argue that long-term modeling of future krill dynamics needs to account for population pressure on resources. In the environmental-driven RK model, this happens automatically, and it is sufficient to use forecasted SAM, ice coverage, and temperature as the controlling parameters. In the BH model, we should additionally consider the dynamics of chlorophyll growth and consumption and use resulting values of chlorophyll and predicted ice coverage, temperature, and SAM as control variables. On the one hand, the first approach may seem somewhat more straightforward, but as the telekinetic relationships between the global SAM index and local environmental conditions vary notably seasonally and geographically (*45*, *51*), it is critical to emphasize that the relationship between SAM and krill recruitment may be specific to the considered region only. On the other hand, the BH model is immediately suited for retrospective modeling or 1-year prognose based on known data on resources and environmental conditions. For example, the BH model, similar to the approach by Veytia *et al.* (*52*), for modeling krill growth potential can easily be applied to simulate krill abundance history across the Southern Ocean based on satellite data.

Our study demonstrates niche separation between larvae and juveniles, when factors favoring larval abundance possibly hamper juvenile survival and vice versa. For example, both models have shown that increased temperature and reduced ice cover during spawning (spring) and initial larval development (summer to early fall) reduce larval losses. In contrast, increased temperature and reduced ice cover may negatively affect juvenile survival. This is most pronounced in spring during the development from the late larval stage (Furcilia VI) to the juvenile stage and in winter before the transition from the juvenile to the adult stage. The reason might be the different physiological demands and different food sources between larval and juvenile krill.

Climate indices also have opposing correlations with larval and juvenile survival: A negative SAM and AAO phase correspond to the success of larvae reproduction and survival, while the success of the juvenile stage can be associated with a positive phase of these indices. This result is close to the findings by Loeb and Santora (*10*), who showed that a positive SOI phase favors larvae, while a negative SOI phase favors juveniles. Such an inverse relationship of climate indices and environmental conditions with larval and juvenile losses can reduce intercohort competition as a year that favors juvenile survival may not promote the development of a large larval cohort and vice versa.

Our study has demonstrated that krill recruitment is substantially affected by chlorophyll concentration, ice coverage, and temperature and that fluctuations in krill biomass are likely caused by competition between adults and larvae for shared resources. We have also found that a model that considers intercohort competition, such as the RK model, provides a better fit to the data with time-constant parameters, while a model that does not account for this competition, such as the BH model, can be more broadly applicable when data on local resource values are available. We also found evidence of niche partitioning between larvae and juvenile krill, with opposing environmental conditions and climate indexes favoring one developmental stage or the other. However, our current model does not account for spatial dynamics, seasonal migrations, and spatial variability of environmental conditions. Accounting for these factors could provide a more detailed and accurate view of the complex interactions between the environment and krill populations, providing a deeper understanding of how climate change and anthropogenic factors affect krill populations and helping to develop effective conservation and management strategies for this critical keystone species.

## MATERIALS AND METHODS

### Data

Data on krill populations have been collected by the PAL-LTER for 27 years (Ross, Steinberg). PAL-LTER stations are located at a distance of 100 km from south to north and about 20 km from west to east, and the selected area is about 400 km by 200 km in size. Only standard stations (−80, −60, ..., 240, 260) were included in our analysis. We used data from the northern and central parts of the grid because the krill population in these areas was monitored throughout the entire period and had coherent spatial population dynamics (*9*). Before determining the mean krill abundance, we cleaned the data and removed 13 outliers (of 1339 samples) with extremely high densities of more than 5000 ind/1000 m^3^. After removing outliers, the median abundance was 7 ind/1000 m^3^, and the mean abundance was 65 ind/1000 m^3^. The row data are available at https://pallter.marine.rutgers.edu/data/.

Most samples of krill abundance were complemented by body length distributions, which allowed us to estimate the percentage of juveniles (≤35 mm) and adults in these samples and to calculate the abundance of each cohort. PAL-LTER data are presented in individuals per cubic meter. To obtain the average abundance of juveniles and adults in the water column, we multiplied their density by the sampling depth. To find the average juvenile and adult abundance, we first averaged the spatial data in increments of 0.5° for longitude and 0.25° for latitude to reduce the weight of stations where multiple samples were taken in the same year. We then found a simple average of adult and juvenile abundance across the entire grid. These data are shown in Fig. 1B.

Unfortunately, PAL-LTER data do not include larval abundance, and we supplemented our dataset with larval abundance in January to February calculated by Loeb and Santora (*10*) based on data from AMLR stations (*24*). Averaged data on krill abundance, krill loss anomalies, and environmental factors are presented in the Supplementary Materials.

#### Chlorophyll and temperature

Chlorophyll and temperature were monitored a few times a month, including winter, at two onshore stations B and E (Fig. 1A). We preferred data from these stations to satellite data because satellite data have only been available since 1997, are less accurate in winter months, and correlate with the monthly averaged data from stations B and E in summer months. Two datasets are available for temperature, with different missing intervals. We combined these sets and first computed the average temperature for each day and then the monthly average temperature. The chlorophyll data are available at https://pallter.marine.rutgers.edu/data/.

#### Ice extent

Ice extent was estimated on the basis of National Snow and Ice Data Center satellite observations, as PAL-LTER data in winter months show maximum ice cover without any interannual difference, but, in summer months, both datasets correlate well with each other. The data were downloaded from https://nsidc.org/data/nsidc-0051/versions/1 using the seaice package for MATLAB (*53*). We included data from the region of 75° to 60° west longitude and 60° to 80° south latitude enclosing the Palmer grid. For each month, we plotted the boundaries of the individual ice-covered regions and then found the total area as the sum of areas of the ice-covered regions.

#### Climate indices

Climate indices provide an overall description of the region climate (Box 1). Climate index anomalies are rapidly oscillating functions, changing sign several times a year, so we used moving averages for the last 9 months, as they revealed the highest correlation with annual anomalies in abundance loss. The indices were obtained from the following sources. SAM index from https://legacy.bas.ac.uk/met/gjma/sam.html, AAO index from www.cpc.ncep.noaa.gov/products/precip/CWlink/daily_ao_index/aao/aao.shtml, SOI from www.cpc.ncep.noaa.gov/data/indices/soi, and Nino 3.4 from https://climatedataguide.ucar.edu/climate-data/nino-sst-indices-nino-12-3-34-4-oni-and-tni.

### Population dynamics models

Three large classes of population models have been proposed to capture krill population dynamics. In decreasing order of complexity, these are integrodifferential, differential, and iterative models. Integrodifferential models most accurately describe the population evolution and body size distribution of individuals (*8*, *54*). However, a spatial generalization of these models is challenging because individuals of the same age at different locations may obtain different weights or other attributes determined by their local history, which greatly complicates modeling of diffusion fluxes. To avoid this problem, differential and iterative models operate with a fixed set of weight or age classes (e.g., larvae, juveniles, and adults). Since the individual traits in each class are fixed, the spatial generalization is usually modeled as diffusive and advective fluxes occurring independently within each class.

Differential models describe a continuous change in abundance in each class in terms of reproduction, mortality, and transitions from younger to older groups at a certain rate. This rate should be chosen such that during the stage developmental time (e.g., 1 year), most individuals would transition to the next stage. This transition process is exponential, which makes these models sensitive to the transition rate. If the rate is too low, then some of the individuals may be delayed in the larval stage, and if the rate is too high, then some of the larvae may reach the adult stage and make an additional contribution to reproduction in the summer of their hatch. Since a single adult krill can produce thousands of larvae, the wrong choice of transition rate can lead to artifacts in reproduction.

Unlike differential models, iterative models assume that at the next iteration, all individuals will either die or move to the next stage, usually associated with their age (*6*, *22*). This approach is simple to implement and does not require transition rate specifications, but it has some other drawbacks. Iterative models compute abundance only at a certain point in time, and all transitions must be strictly synchronized (e.g., occurring in summer). This makes it difficult to explicitly model processes whose intensity varies continuously throughout the year, for example, the presence of predators in summer and their absence in winter or seasonal changes in fishing intensity.

### The life history labyrinth model

#### Krill population model with time-constant parameters

Here, we propose a hybrid approach for modeling age structured populations that combines the advantages and avoids the disadvantages of the other model classes. We describe the population dynamics with differential equations but assume that a transition from one stage to another is only allowed during the time interval τ when the transition window is open (Fig. 2A). The advantage of our approach over conventional differential models is that we can always arrange the transition windows to block passages from stage *n* − 1 to stage *n* + 1 without completely passing through stage *n*, for example, from larvae to adults, bypassing the juvenile stage. As a result, we can build a model with a weak dependence on the transition rate *r*, if it is large enough that most individuals move to the next stage in the transition time τ. Since the transition process is exponential, the proportion of individuals that fail to move is ρ = *e*^−*r*τ^. Setting *r* = 30 year^−1^, τ = 0.2 year in our model (see Table 1), we obtain ρ = 0.002.

The function activating transition windows is defined as

$$f(t,t_{0})=\exp\left(-{\left\{\frac{\sin[\pi \;(t-t_{0})]}{\sin(\pi \tau /2)}\right\}}^{2m}\ln2\right)$$

where *t*, *t*_0_, and τ are expressed in years. This is a smooth dome-shaped function with period 1, approaching 1 at time points *t*_0_ + *i* (*i* is integer) when the transition is open during time interval τ and approaching 0 when the transition is closed. With increasing exponent *m*, the shape of the function changes from a Gaussian to a rectangular pulse (we used, *m* = 5). The normalizing factor ln2 in the exponent is chosen from the condition $\int_{0}^{1}f(t)dt\underset{m\to \infty }{\to }\tau$. In the model, we used three activating functions (Fig. 2C): activation of egg production in summer, *f*_sum_(*t*) = *f*_act_(*t*,0), transition of larvae into the juvenile stage in spring, *f*_spr_(*t*) = *f*_act_(*t*,9/12), and transition of 1.5-year-old juveniles into the adult stage, *f*_wnt_(*t*) = *f*_act_(*t*,1/2).

Reproduction is defined by a modified Deriso-Schnute generalized recruitment function (*55*, *56*).

$$R(A)=L_{\max}\;A\;{\left(1-\frac{L_{\max}\beta_{\gamma }}{G_{\max}}A\right)}^{\frac{1}{\gamma }}$$

where *L*_max_ is the initial slope of the recruitment curve (number of eggs hatched per unit of time per adult in the absence of competition), *G*_max_ is the maximum number of eggs hatched (habitat capacity), and the parameter γ controls the smooth transition from the BH model at γ = −1 to the RK model at γ → −0 (Fig. 2B). The normalizing constant β_γ_ depends only on the controlling parameter, β_γ_ = γ/(γ + 1)^(1+1/γ)^. This dependence is equivalent to the canonical form proposed by Schnute (*55*) but makes it easier to compare RK and BH models, since, here, the maximal habitat capacity, *G*_max_, is an independent parameter, while the maximum of the canonical form *R*(*A*) is nonlinearly linked to the initial slope of the function.

The simulation results for parameter values attained from a least-squares fit of this model to the data are shown in Fig. 3, by the green lines. We denote these time-independent parameters as “standard parameters” and this model as a time-constant model (although the transition rates are functions of time) to distinguish it from a model in which the parameter values change annually.

#### In the annually varying model

We additionally introduced annual anomalies δ*_Y_* determining deviations of mortality for the cohort that spent most of its lifetime in year *Y*. Larval and juvenile mortality in year *Y* was determined by the anomalies of their abundance losses δ_L,*Y*_ and δ_J,*Y*_, as $m_{L,Y}^{\prime }=m_{L}\;\exp\;\delta_{L,Y}$ and $m_{J,Y}^{\prime }=m_{L}\;\exp\;\delta_{J,Y}$, where *m*_L_ and *m*_J_ are the larval and juvenile mortality rates of the time constant model. To align the mortality changes to the lifespan of these cohorts (see Fig. 2A), we applied δ_L,*Y*_ in the interval from 1 November of year *Y* − 1 to 31 October of year *Y* and δ_J,*Y*_ from 1 August year *Y* − 1 to 31 July year *Y*. We also considered models with anomalies of adult losses, but, in most cases, the values obtained by fitting those anomalies did not deviate significantly from zero, so we do not include these results. The source code for the simulation is publicly available at (*57*).

### Fitting the model

The population dynamics of the time-constant models are determined by the initial abundances and five standard parameters: mortality in each cohort and the initial slope and maximum value of the reproduction curve. We fitted the parameters of the time-constant models by minimizing the weighted SD between the logarithms of data and modeled abundance of adults, juveniles, and larvae on 1 January (see Table 1 for fitted parameters).

Namely, we minimized the loss function with regularization of fitted parameters

$$\mathrm{Cost}=\frac{1}{n}\sum_{Y=1}^{n}[\alpha_{L}{\left(\log L_{Y}^{\prime }-\log L_{Y}\right)}^{2}+{\left(\log J_{Y}^{\prime }-\log J_{Y}\right)}^{2}+\alpha_{A}{\left(\log A_{Y}^{\prime }-\log A_{Y}\right)}^{2}]+\lambda \sum_{k}\;{\left(\frac{\theta_{k}-\theta_{k0}}{\theta_{k0}}\right)}^{2}$$

Here, *n* = 27 is the number of observations, *Y* is the observation year, and *k* runs over all fixed parameters θ*_k_* to be optimized. Symbols with a strike denote data values and without it the model results. The first term in the cost function is the mean square deviation of the logarithms of abundances in the model results (as of 1 January) and observations. We used weight α_A_ = 2 for adults and α_L_ = 2/3 for larvae to equalize the cohort abundance scales. The second term gives an additional penalty for deviations of model parameters θ*_k_* from their prior values θ_*k*0_ (table S1), which, in the unperturbed model, were chosen on the basis of literature data, see, e.g., the choice of similar parameters in (*8*). The regularization constant λ = 0.01, was chosen so that the weighted relative squared deviation of the model parameters from their prior values was approximately 5% of the value of the entire cost function. With this choice, the fitted parameters were only weakly dependent on the prior values.

To find the minimal necessary anomalies, we again used the regularization technique, meaning that we minimized the weighted sum of the anomaly’s variance and the model-data variance. We minimized the following cost function

$$\mathrm{Cost}=\frac{1}{n}\sum_{Y=1}^{n}[\alpha_{L}{\left(\log L_{Y}^{\prime }-\log L_{Y}\right)}^{2}+{\left(\log J_{Y}^{\prime }-\log J_{Y}\right)}^{2}+\alpha_{A}{\left(\log A_{Y}^{\prime }-\log A_{Y}\right)}^{2}]+\lambda_{r}\sum_{Y}\;[\delta_{L,Y}^{2}+\delta_{J,Y}^{2}]$$

The last term in the cost function provides a penalty for the high mean square value of the loss anomalies. The penalty is determined by the regularization constant λ*_r_*. Too small value of λ*_r_* leads to model overfitting, because the annual anomalies can obtain too large deviations, which does not allow to generalize the data. Too large λ*_r_* leads to small loss anomalies and poor model fit. In both cases, the resulting anomalies are poorly predicted by environmental conditions. We performed the fit with regularization constants λ*_r_* equal to 0.001, 0.003, 0.01, 0.03, and 0.1. The results are presented for λ*_r_* = 0.01, at which the number of significant correlations in Fig. 5 reached its maximum. In addition to the anomaly values, we also fitted the initial values in each cohort but did not change the standard model parameters of the time-constant model.

Determining the loss anomalies is a challenging optimization problem in a high-dimensional space, as we need to fit 54 values defining the larval and juvenile anomalies for 27 years. To address this, we used a MATLAB package for robust optimization (*58*) to efficiently search for the minimum of such a function.

To localize the range of parameters confining the global minimum, we performed 300 primary minimization cycles, shifting the search area so that the combination of parameters found in the previous cycle was in the middle of the search area for the next cycle. Each minimization cycle involved approximately 30,000 model calculations. Saving computation time, this approach allows the search window to slide in the direction of the global minimum.

When the position of the search window stabilized, we conducted additional 300 independent minimization cycles, each comprising around 30,000 model calculations. All combinations of anomalies found during the 300 minimization cycles provide a very good model-to-data fit (Fig. 3, blue lines) with less than 2% change in the mean square deviation of the model from data. The anomalies providing the best approximation (used for plotting the blue lines in Fig. 3) are highlighted as red dots in Fig. 4; the anomalies, obtained in other minimization cycles (blue dots, Fig. 4), characterize the confidence interval. We interpret an anomaly as significantly deviating from zero (filled circle), when at least 95% of its values are on one side of zero. A nonsignificant anomaly (open circle) means that at least in 5% of the minimization cycles, we found an anomaly of the opposite sign without noticeably degrading the quality of the data fit.

### Fused LASSO regression and *K*-fold cross-validation

Traditional feature selection algorithms aim to remove irrelevant variables and identify an optimal (e.g., based on Akaike information criterion values) set of variables predicting the target variable. However, these approaches may introduce a bias in feature selection by prioritizing, for example, the influence of chlorophyll values in 1 month and discarding the influence of correlated chlorophyll values in the following month. Another option is to use aggregated variables, such as mean annual chlorophyll levels (*3*) or mean winter ice coverage (*6*). However, the choice of aggregation range is somewhat arbitrary and again does not allow for the timing of the influence of different factors. Consequently, although these methods facilitate the development of predictive models, they provide only limited insight into the dynamics of the influence of environmental factors during krill life cycle.

To address this problem with a more flexible method, we used fused LASSO regression (*28*, *29*). This machine learning technique offers an efficient solution for linear regression when some explanatory variables, such as consecutive monthly chlorophyll levels, are correlated with each other and are assumed to have a similar effect on the target variable.

In conventional linear regression, *h*(*X*) = ∑ *a_j_x_j_*, we minimize the cost function, $J=\frac{1}{2m}\sum_{i}{\left[Y_{i}-h(X_{i})\right]}^{2}$, expressing the mean square deviation between model and data. The fused LASSO regression additionally minimizes the values of the regression coefficients and the differences between adjacent regression coefficients. We use the following cost function [this variant is also referred as joint LASSO regression (*29*)]

$$J(\text{Θ})=\frac{1}{2m}\sum_{i}{\left[Y_{i}-h(X_{i})\right]}^{2}+\lambda \sum_{j}|a_{j}|+\tau \sum_{F}\sum_{i_{F}}{\left(a_{i_{F}}-a_{i_{F}-1}\right)}^{2}$$

In this cost function, the first term refers to the deviation between the linear model and the data. The second term regularizes the regression by penalizing nonzero coefficients, which, as in conventional LASSO regression, allows for sparse linear models with some coefficients set to zero. The last term, specific to fused LASSO, adds a penalty for differences between adjacent coefficients within each group *F* of environmental factors (temperature, chlorophyll, ice cover, and SAM). Index *i_F_* enumerates the coefficients corresponding to the monthly values of factor *F*. This penalty increases as the difference between the coefficients in successive months increases.

For a small value of λ, the model is as flexible as possible, approaching ordinary linear regression with most coefficients *a_j_* ≠ 0. By increasing λ, we increase the penalty and can end up with a “linear” model that predicts only a constant value *a*_0_. Similarly, for small values of τ, the penalty for the difference *a_i_F__* − *a*_*i_F_*−1_ becomes minimal and we obtain a simple LASSO regression, whereas large values of τ will lead to a model in which the coefficients *a_i_F__* within each group achieve the same values.

Choosing the optimal hyperparameters λ and τ involves finding a balance between the flexibility of the model and its ability to generalize the data. To find the point of this balance, we perform regression on a training subset of data with different combinations of λ and τ, and find these values λ_0_ and τ_0_ that the resulting regression model produces minimal error on a validation subset that was not used to fit the model. For large datasets, the traditional approach allocates 60% of the data to model training, 20% to model validation, and 20% for testing results. For small datasets, the most effective method is *K*-fold cross-validation. In this approach, the data are divided into *K* segments or “folds.” For each λ and τ, the regression is performed *K* times, using *K* − 1 folds to fit the model and the remaining fold to find the validation error. This yields *K* validation errors, which are averaged to obtain the cross-validation error. The parameters λ_0_ and τ_0_ are found from the minimum cross-validation error condition. In situations where the predictors have no effect on the response variable, the regression model’s prediction for the validation data is usually worse than just the mean of the response. In these cases, the cross-validation error consistently decreases as λ increases, indicating that the response cannot be accurately predicted by any linear combination of these environmental factors. To perform fused LASSO regression we used package fusedLASSO (*59*).

To get an overview of the importance of different environmental factors for specific loss anomalies, we performed the hyperparameter optimization and fitted fused LASSO models with different combinations of drivers, from models including one environmental factor to models with four environmental factors, totaling 15 models for each set of loss anomalies, δ_*k*,*Y*_. In LASSO regression, the magnitude of the coefficient $a_{i_{F}}^{k}$ determines the explanatory power of the corresponding variable. Therefore, we can estimate the importance of factor *F* in explaining loss anomalies in cohort *k* as the average of the absolute values of $a_{i_{F}}^{k}$ across all the 15 models.

Consistent with the results of our correlation analysis, we found that in the BH model, chlorophyll concentrations mainly determine larval loss anomalies, with other factors playing a minor role (fig. S1). Conversely, larval loss anomalies in the RK model are mainly explained by the SAM index, with smaller contributions from other factors. For juvenile losses, temperature acts as an important factor in both models, while ice also plays an important role in the RK model. Chlorophyll and SAM values have less power in explaining the anomalies of juvenile losses compared to their influence on larval losses.

Last, of the 15 fused LASSO regression models for each set of loss anomalies, we selected one model with the lowest cross-validation error and, if the difference in validation error between the models was less than 2%, the one that included the minimum number of environmental factors. This model was used for Fig. 6.
