# Supplementary material for: Driving forces of Antarctic krill abundance
Source: Sci Adv. 2023 Dec 15;9(50):eadh4584. doi: 10.1126/sciadv.adh4584 (PMC10848738; doi:10.1126/sciadv.adh4584)
Supplement: Supplementary file 1 — Figs. S1 and S2 Table S1 Legends for data S1 to S4 [file sciadv.adh4584_sm.pdf]

Science Advances
AAAS

Supplementary Materials for

Driving forces of Antarctic krill abundance

Alexey Ryabov *et al.*

Corresponding author: Alexey Ryabov, alexey.ryabov@uol.de; Bettina Meyer, bettina.meyer@awi.de

Sci. Adv. **9**, eadh4584 (2023)
DOI: 10.1126/sciadv.adh4584

The PDF file includes:

Figs. S1 and S2
Table S1
Legends for data S1 to S4

Other Supplementary Material for this manuscript includes the following:

Data S1 to S4

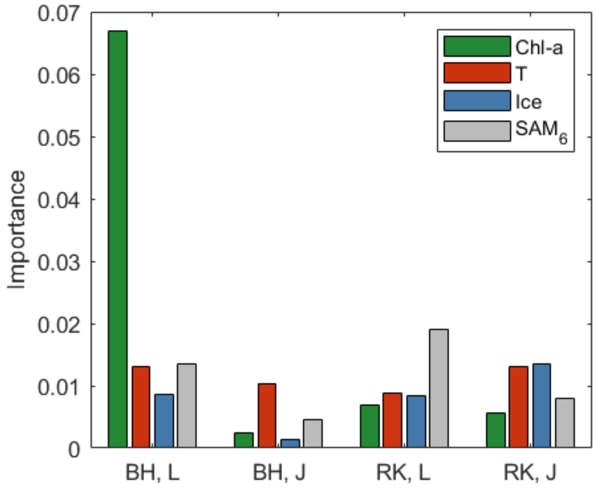

Fig. S1. Relative importance of four primary environmental factors in explaining deviations in loss rates of larvae (L) and juveniles (J) in the BH and RK-models. Note that importance equals the means absolute value of the fused LASSO regression coefficients and does not indicate whether the factor is negatively or positively related to the loss anomaly.

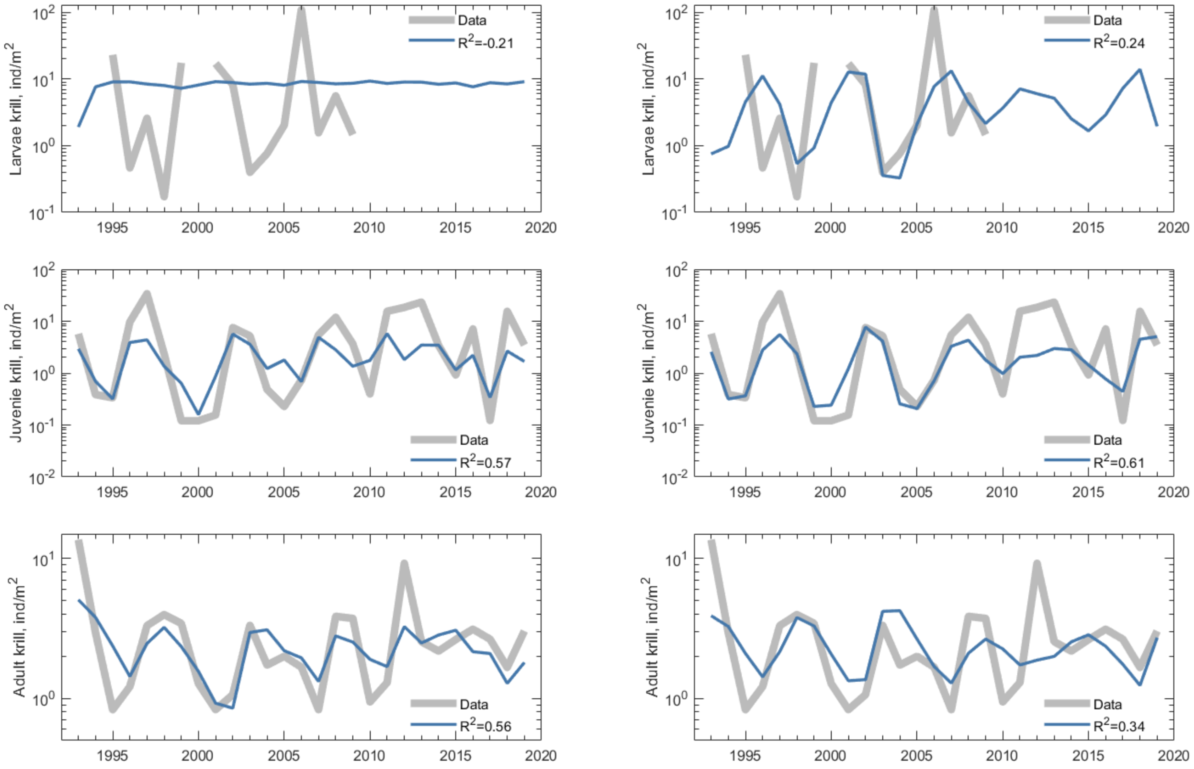

Fig. S2. Projection of krill dynamics by environmental-driven RK and BH-models for larvae (top), juveniles (middle) and adults (bottom). The abundance data for each cohort (grey) are compared with the output of the BH-model (left) and the RK-model (right) in which anomalies in abundance loss are modulated by environmental factors following linear models obtained by fused LASSO regression (Fig. 6, main text).

L_{max}	2500	eggs year^{-1} ind^{-1}	Maximal number of larvae produced by one adult per time unit
G_{max}	450	eggs year^{-1} m^{-2}	Maximal number of larvae per square meter per time unit
m_L	2.25	year^{-1}	Larval mortality
m_J	1.4	year^{-1}	Juvenile mortality
m_A	0.8	year^{-1}	Adult mortality
**Prior values for the initial density on 01.01.1993 for the time-constant model**			
L_0	5	ind/m^2	Larvae
J_{10}	5	ind/m^2	Juveniles
A_0	11	ind/m^2	Adults

Table S1. Prior values for fitting of parameters

Separate data files

Data S1.
Monthly average environmental factors.

Data S2.
Average abundance of larvae, juvenile and adult krill.

Data S3.
Loss anomalies calculated for Beverton-Holt model.

Data S4.
Loss anomalies calculated for Ricker model.
